# Depression, anxiety disorders, and metabolic syndrome in a population at risk for type 2 diabetes mellitus

**DOI:** 10.1002/brb3.306

**Published:** 2015-01-21

**Authors:** Kai G Kahl, Ulrich Schweiger, Christoph Correll, Conrad Müller, Marie-Luise Busch, Michael Bauer, Peter Schwarz

**Affiliations:** 1 Department of Psychiatry and Psychotherapy, Carl-Gustav-Carus UniversityTU Dresden, Germany; 2 Department of Psychiatry, Social Psychiatry and Psychotherapy, Hannover Medical SchoolHannover, Germany; 3 Department of Psychiatry and Psychotherapy, UK-SH, Lübeck University Medical SchoolLübeck, Germany; 4 The Zucker Hillside Hospital, Psychiatry Research, North Shore-Long Island Jewish Health SystemGlen Oaks, New York; 5 Department of Internal Medicine, Carl-Gustav-Carus UniversityTU Dresden, Germany

**Keywords:** Anxiety disorders, major depressive disorder, metabolic syndrome, mood disorders, type 2 diabetes mellitus

## Abstract

**Background:**

Depressive symptoms have been associated with type 2 diabetes mellitus (T2DM), but less is known about anxiety disorders that can be comorbid or exist without depression.

**Methods:**

We evaluated the prevalence of psychiatric disorders in subjects consecutively examined at an outpatient clinic for diabetes prevention who were at-risk for T2DM, defined by FINDRISK scores, and compared metabolic syndrome (MetS) frequencies between subjects with and without psychiatric morbidity, entering also relevant variables for MetS into multivariate analyses. All subjects underwent an oral glucose tolerance test (OGTT). Psychiatric diagnosis was confirmed using a Structured Clinical Interview for DSM-IV.

**Results:**

Of 260 consecutively screened subjects, 150 (56.9 ± 8.1 years old, males = 56.7%, BMI = 27.2 ± 4.1 kg/m^2^) were at-risk for T2DM and were included. MetS, present in 27% of males and 25% of females, was significantly associated with having a current anxiety disorder (*P* < 0.001) and lifetime major depression (*P* < 0.001). In logistic regression analysis, MetS was significantly associated with lifetime major depression, presence of any anxiety disorder, body weight, and physical activity.

**Conclusions:**

Our data in a high-risk group for T2DM support the association between depressive disorders and MetS, pointing to a similar role of anxiety disorders. Screening for anxiety and depression is recommended in this group at risk for T2DM.

## Introduction

Depressive disorders and anxiety disorders are highly prevalent. In the general population, the 12-month prevalence rates are approximately 18% for anxiety disorders and 10% for mood disorders (Kessler et al. 2005). Both depressive and anxiety disorders have an important general impact on role disability (Merikangas et al. 2007).

Diabetes mellitus, type 2 (T2DM) has a prevalence around 8% in middle aged men and women in Germany (Schipf et al. 2012). There is a strong association between depression and incidence of type 2 diabetes and a weaker relation between diabetes and risk of depression (Mezuk et al. 2008). Depression has been described as a major risk factor for the development of type 2 diabetes with a 1.6-fold risk as assessed by a meta-analysis (Arroyo et al. 2004), and is established as an independent risk factor for cardiovascular disease and overall mortality (Wulsin et al. 1999; Ferketich et al. 2000). Comorbid depressive disorders magnify the impact of chronic physical illness (Stein et al. 2006). The prevalence of depressive or anxiety disorders is approximately 60% higher in patients with type 2 diabetes than in the general population (Ali et al. 2006; Fisher et al. 2008; Collins et al. 2009). The prevalence of depression is already higher in men who are unaware of suffering from diabetes mellitus (Gale et al. 2010). Depressive symptoms are more common in women with impaired glucose metabolism as assessed by glucose tolerance test than in the general population, but not in men (Adriaanse et al. 2008).

The metabolic syndrome (MetS) refers to a cluster of symptoms that increase the risk for morbidity and mortality from cardiovascular disease, type 2 diabetes mellitus, and all-cause mortality (Lakka et al. 2002). Cross-sectional studies examining the association between depression, anxiety, and the MetS yielded conflicting results, either pointing to a general association (Heiskanen et al. 2006; Skilton et al. 2007; Dunbar et al. 2008; Miettola et al. 2008; Heppner et al. 2009; Kahl et al. 2012; Pan et al. 2012; Vancampfort et al. 2013), a gender-specific association (Kinder et al. 2004; Gil et al. 2006; Toker et al. 2008; Viinamaki et al. 2009; Capizzi et al. 2010) or no association (Herva et al. 2006; Hildrum et al. 2009; Carpiniello et al. 2011; Kimura et al. 2011). A recent study found an association for depressive, but not anxiety symptoms with MetS (Tziallas et al. 2011). The results of prospective studies suggest that depressive symptoms predispose females to the development of MetS (Raikkonen et al. 2007; Goldbacher et al. 2009; Vanhala et al. 2009) and that the metabolic syndrome or some factors of its underlying pathophysiology predispose to depression (Akbaraly et al. 2009).

Only few studies have addressed the association of depressive disorders or anxiety disorders on the one hand and the MetS on the other in high-risk populations. Skilton and coworkers identified an association between MetS and depression scores, but not anxiety scores, in a population referred on the basis of possessing at least one traditional cardiovascular risk factor (Skilton et al. 2007).

The relationship between risk factors and pathology may vary in different populations. There may be ceiling effects or floor effects. Since high-risk populations make up the majority of patients in medical treatment, the association of risk factors with pathology is of particular importance for planning interventions, whereas studies in the general population have higher relevance for the development of prevention.

Therefore, we assessed patients at risk for the development of type 2 diabetes, defined by the FINDRISK score (Ford and Giles 2003; Lindstrom and Tuomilehto 2003), who were consecutively examined at a specialized outpatient clinic for diabetes prevention for MetS and psychiatric diagnoses of depression or anxiety disorders. Based on prior data, we hypothesized that there would be a higher prevalence of MetS in subjects with a current or lifetime diagnosis of major depressive disorder.

## Materials and Methods

### Participants

Men and women at risk for the development of type 2 diabetes, defined by the FINDRISK score (Ford and Giles 2003; Lindstrom and Tuomilehto 2003), consecutively examined at a specialized outpatient clinic for diabetes prevention at the Technical University of Dresden, Germany, were included. Patients were assessed using standardized interviews for mental disorders and by obtaining anthropometric and metabolic profiles based on oral glucose tolerance test. The FINDRISK score is a screening tool for identifying high-risk subjects in the population, and is a standard application to subjects visiting the outpatient clinic for diabetes prevention at the Technical University of Dresden. The study was approved by the local ethics committee of the TU Dresden, and all participants gave their written informed consent prior to the beginning of the study. The subjects derived from German families with a family history of type 2 diabetes, obesity, or dyslipoproteinemia. Exclusion criteria were: diagnosed diabetes mellitus, severe renal disease, disease with a strong impact on life expectancy, and therapy with drugs known to influence glucose tolerance (thiazide diuretics, beta blockers, steroids, psychopharmacologic medications, particularly antipsychotics, tricyclic antidepressants, or serotonin reuptake inhibitors).

### Measures

Metabolic syndrome was defined according to the criteria of the National Cholesterol Education Adult Treatment Panel III-R (NCEP ATP III; Ford and Giles 2003), that is, (1) waist circumference >102 cm in men and >88 cm in women; (2) hypertriglyceridemia ≥150 mg/dL; (3) low HDL cholesterol <40 mg/dL in men and <50 mg in women; (4) high blood pressure ≥130/85 mmHg; and (5) high fasting glucose ≥110 mg/dL.

Psychiatric diagnoses were confirmed by face-to-face interviews using the Structured Clinical Interview for DSM-IV mental disorders (Wittchen et al. 2006). The Beck depression Inventory (BDI-2; Hautzinger et al. 2006) was used to determine the extent of depressive symptomatology. Lifestyle variables were assessed by interview: Smoking habits were expressed as pack-years (cigarettes per day × years of smoking/20). Physical activity was determined with a 6-point Likert scale, with descriptors ranging from never (1 point) to very often (6 points). Alcohol consumption was expressed as drinks per day (equivalents of 14 g alcohol). Social status was assessed by asking for professional status.

All individuals underwent a 75 g oral glucose tolerance test following an overnight period of fasting (10 h minimum) with measurement of metabolic syndrome parameters, fasting plasma glucose, insulin, and C-peptide at fasting and at 30, 60, 90, and 120 min after glucose challenge. The screening information in the FINDRISK about exercise and consumption of vegetables, fruit, or whole wheat products was not used as outcome variable.

### Statistical analyses

We performed *χ*²-tests for the comparison of MetS rates between subjects with and without psychiatric morbidity, and repeated measurements analysis of variance (ANOVA) for group comparisons concerning the oral glucose tolerance tests. Sphericity of the data was assessed using Mauchly's test. In the case of violation, Greenhouse-Geisser correction was used. A binary logistic regression analysis was performed to determine factors associated with MetS, and a stepwise multiple regression analysis was conducted to identify independent variables associated with the number of fulfilled criteria for MetS, correcting for body weight and physical activity (SPSS 20.0; IBM software, Ehningen, Germany).

## Results

Of 260 subjects consecutively examined at a specialized outpatient clinic for diabetes prevention at the Technical University of Dresden, Germany, who were screened using the FINDRISK, 158 were eligible. 150 subjects (85 men, 65 women) gave informed consent for the study and underwent full study procedures. The average age of the subjects was 56.9 ± 8.1 years; average BMI was 27.2 ± 4.1 kg/m^2^. None of the subjects received any psychotropic treatment. Altogether, 23 of the 85 male participants (27%) and 16 of the 65 female participants (25%) fulfilled MetS criteria. A total of 28 subjects (18.7%) had a lifetime diagnosis of major depressive disorder but no current major depression, 10 had current major depressive disorder (6.7%), and 35 (23.3%) had at least one anxiety disorder (nine panic disorder, 13 social phobia, two general anxiety disorder, one obsessive compulsive disorder, eight posttraumatic stress disorder). Twelve participants (8.0%) were diagnosed with both major depression and an anxiety disorder. Subjects with depression or anxiety disorders had similar occupational status, age, weight, height, BMI, smoking habits, alcohol intake, and physical activity as subjects without mental disorders. Also waist circumference, blood pressure, fasting glucose, triglycerides, and HDL cholesterol were not significantly different (Table 1).

**Table 1:** Anthropometric and sociodemographic data in patients at risk for type 2 diabetes mellitus. Please note that due to comorbidity patients with anxiety and depression (current or lifetime) may be represented in several columns

	Control group (*N* = 90)	Anxiety disorders (*N* = 35)^1^	Lifetime MDD (*N* = 28)	Current MDD (*N* = 10)
Female (*N*; %)	33 (35)	20 (57)	17 (61)	7 (70)
Occupational status (%)				
White collar	21.1	31.4	32.1	30
Blue collar	24.4	34.3	32.1	20
Unoccupied	4.4	5.7	3.6	20
Pension	50	28.6	32.1	30
Age (years)	52.3 ± 14.9	51.4 ± 13.1	51.6 ± 13.0	51.3 ± 14.7
Weight (kg)	82.8 ± 15.8	84.1 ± 15.8	80.5 ± 12.3	86.9 ± 25.1
Height (m)	1.7 ± 0.1	1.7 ± 0.1	1.7 ± 0.1	1.7 ± 0.1
BMI	27.2 ± 3.8	28.6 ± 4.2	27.7 ± 3.2	30.0 ± 6.8
WC (cm)	104.9 ± 8.1	107.7 ± 8.5	105.9 ± 6.7	109.4 ± 11.0
Systolic BP (mmHg)	132.7 ± 17.9	143.4 ± 21.6	137.7 ± 18.1	133.6 ± 15.7
Diastolic BP (mmHg)	79.3 ± 11.7	82.8 ± 10.6	84.6 ± 10.2	78.9 ± 11.7
FG (mmol/L)	5.1 ± 0.6	5.2 ± 0.8	5.3 ± 0.8	4.9 ± 0.6
Triglycerides (mmol/L)	1.6 ± 1.5	2.0 ± 1.5	1.9 ± 1.1	1.4 ± 0.5
HDL-C (mmol/L)	1.5 ± 0.4	1.5 ± 0.4	1.5 ± 0.4	1.4 ± 0.2
Number of MetS criteria	1.4 ± 1.2	2.2 ± 1.4	2.3 ± 1.5	1.9 ± 1.2
MetS (*N*, %)	14 (16)	17 (49)*	12 (37.5)*	4 (40)
Smoking (pack-years)	5.1 ± 9.8	3.2 ± 5.7	3.8 ± 6.9	7.7 ± 14.9
Alcohol intake (drinks per week)	3.9 ± 4.9	4.1 ± 5.3	4.0 ± 4.8	3.2 ± 4.8
Physical activity	3.1 ± 1.7	3.2 ± 1.8	2.8 ± 1.9	3.8 ± 1.7
BDI (sum score)	3.1 ± 3.8	6.8 ± 6.4	6.0 ± 6.2	14.9 ± 7.6

AD, anxiety disorders; BDI, Beck depression inventory; BMI, body mass index; CG, comparison group; diastBP, diastolic blood pressure; systBP, systolic blood pressure; FG, fasting glucose; HDL, high-density cholesterol; cMDD, current major depressive disorder; ltMDD, lifetime major depressive disorder; MetS, metabolic syndrome; WC, waist circumference.

The MetS was significantly more frequent in subjects with anxiety disorders (AD) and with lifetime major depressive disorder (ltMDD). We observed a trend toward increased rate of the MetS in subjects with current MDD.

* Indicates a significant difference compared to CG (*P* < 0.05).

1 Anxiety disorders (comorbidities are possible): nine panic disorder, 13 social phobia, two general anxiety disorder, one obsessive compulsive disorder, eight posttraumatic stress disorder.

We observed a higher frequency of MetS in subjects with current anxiety disorders (ADs) compared to subjects without ADs and major depressive disorder (MDD; *χ*² = 17; df = 1; *P* < 0.001), and a trend toward increased MetS in subjects with current MDD (*χ*² = 3.6; df = 1; *P* = 0.08; Table 1). Furthermore, the MetS was significantly more frequent in subjects with a lifetime history of MDD (*χ*² = 14.2; df = 1; *P* < 0.001; Table 1).

A binary logistic regression model with MetS as the dependent variable showed a positive association with lifetime major depression, presence of any anxiety disorder, weight, and physical activity (Table 2). Gender, age, height, pack years of smoking, current MDD, and reported alcohol consumption were not significantly associated. The overall model chi square was 36.5, *P* < 0.001; Nagelkerke's *R*^2^ = 0.33 is in the low to moderate range. When subjects with posttraumatic stress disorder (PTSD) were excluded from the analysis, lifetime major depression, any anxiety disorder, weight, and current major depression were associated with the MetS (data not shown).

**Table 2:** Association of lifetime depression and anxiety disorders with the metabolic syndrome. Results of the binary logistic regression analysis

	ß	Wald	df	*P*	eß (odds ratio)	eß range
AD	1.7	11.7	1	>0.01	5.8	2.1–15.7
Weight (kg)	0.1	5.9	1	0.02	1.1	1.0–1.1
Lifetime MDD	1.2	4.6	1	0.03	3.3	1.1–9.8
Physical activity	−0.3	4.6	1	0.03	0.7	0.6–1.0
Alcohol intake (drinks per week)	0.02	0.2	1	0.67	1.0	0.9–1.1
Smoking (pack-years)	0.01	0.1	1	0.75	1.0	0.9–1.0
Gender	0.1	0.03	1	0.86	1.1	0.3–4.9
Height (m)	2.8	0.5	1	0.48	15.9	0.0–∞
cMDD	0.6	0.4	1	0.41	1.8	0.3–11.9
Age (years)	0.01	0.02	1	0.71	1.0	1.0–1.0

AD, any anxiety disorder; cMDD, current major depressive disorder; ltMDD, lifetime major depressive disorder.

Current anxiety disorders (AD) and lifetime major depression (ltMDD) are associated with the metabolic syndrome according to NCEP ATP-3 criteria. Furthermore, high weight and low physical activity are associated with the metabolic syndrome.

A stepwise multiple regression analysis with the number of positive criteria for MetS (systolic and diastolic blood pressure, fasting glucose, waist circumference, triglycerides, and HDL-cholesterol) as the dependent variable showed significant effects of weight, lifetime depression (MDD), height, physical activity, any anxiety disorder (AD), and age as independent variables (Table 3). The overall model *R*^2^ = 0.32.

**Table 3:** Predictors of the number of metabolic syndrome factors. Stepwise multiple regression analysis

	Regression coefficient ß	ß range	*R*^2^ change	Significance
Weight (kg)	0.05	0.03–0.06	0.17	<0.01
ltMDD	0.70	0.24–1.15	0.06	<0.01
Height (m)	−2.7	−5.0 to −0.39	0.04	0.02
Physical activity	−0.12	−0.22 to −0.02	0.03	0.02
AD	0.49	0.07–0.91	0.02	0.02
Age (years)	0.02	0.00–0.03	0.02	0.03

AD, anxiety disorders; ltMDD, lifetime major depressive disorder; cMDD, current major depressive disorder.

Not included in the model were alcohol intake, smoking, and cMDD.

A repeated measures ANOVA model did not reveal any significant effects of depression or anxiety on the time course of glucose, insulin or C-peptide during the oral glucose tolerance test after correction for weight and physical activity. Only the covariate weight showed significant effects on all three parameters.

## Discussion

Our data indicate that lifetime major depression and current anxiety disorders are independently associated with the MetS in a population at high risk for type 2 diabetes mellitus. This association was independent of gender and was maintained after correction for age, body weight, and several life style variables. It was also maintained after exclusion of subjects with PTSD. The finding is in accordance with studies demonstrating an association between depression and the metabolic syndrome in the general population (Kinder et al. 2004; Skilton et al. 2007). Whereas the relationship between depression, metabolic syndrome, and T2DM is well established, there are less data on anxiety disorders. Our results suggest that anxiety disorders may be independently associated with MetS. Due to limitations of statistical power, we were not able to localize these associations to one or several specific subtypes of anxiety disorders or specific components of MetS. MetS tended toward being more frequent in subjects with current depression compared to subjects without depression or anxiety disorders.

Factors that have been discussed to underlie the association between depression and cardiovascular disorders comprise increased activity of the HPA-system (Vreeburg et al. 2009), a disbalance of the immune system (Miller et al. 2009), risk taking, and a lifestyle characterized by low exercise especially in adolescents and young adults with depression (Ruchkin et al. 2006; Strohle et al. 2007), increased volumes of visceral fat (Kahl et al. 2005), and low glucose disposal rates (Schweiger et al. 2008). Current and remitted depression did not differ in the degree of the activation of the hypothalamic-pituitary-axis in a large population-based study (Vreeburg et al. 2009). Furthermore, some antidepressants have been implicated in weight gain and the development of insulin resistance (Chokka et al. 2006; Kozumplik and Uzun 2011). However, subjects in our study were not treated with antidepressants.

Our data similar to other studies do not support the notion that the association can be explained exclusively by differences in lifestyle variables. However, we should emphasize that not all relevant lifestyle variables were considered in the study and that the assessment of lifestyle variables is based on patient report with limited reliability. Due to the cross-sectional nature of our data, we cannot make any statements about the direction of the relation between depression and anxiety and MetS or diabetes. Interestingly, prospective cohort studies found the MetS also predictive for the development of depressive symptoms and depressive disorders (Akbaraly et al. 2009; Takeuchi et al. 2009). Taken together, results from several studies point to a bidirectional relationship of depression and the MetS.

Few studies have been undertaken on the association of anxiety disorders with MetS (Skilton et al. 2007). Heppner and colleagues examined 253 male and female veterans and found an increased prevalence of the MetS in patients with PTSD, in particular in those with severe PTSD (Heppner et al. 2009). In a cross-sectional study by Carroll and colleagues, generalized anxiety disorder was positively associated with the MetS (Carroll et al. 2009). Interestingly, data from the Netherlands Study of Depression and Anxiety (NESDA) showed an association between anxiety and components of the MetS using a dimensional approach. In particular, waist circumference, triglycerides, and blood pressure were strongly associated with anxiety (van Reedt Dortland et al. 2010). Similar to findings in major depressive disorder, a dysregulation of cortisol secretion has been found in anxiety disorders (Vreeburg et al. 2010). In accordance with these clinical data, our results show an association of anxiety disorders with MetS in a population that is, already at risk for the development of type-2 diabetes mellitus.

The results from this study need to be interpreted within several limitations. These include the relatively small sample size, precluding separate analyses of individual anxiety disorders, and likely underpowered analyses of the influence of current major depression (*n* = 10). In addition, the absence of significant group differences in anthropometric data and factors determining the metabolic syndrome (blood pressure, fasting glucose, triglycerides, HDL cholesterol) may suggest type 2 error due to insufficient power. The study does not allow an exact estimation of the prevalence of mental disorders in a high-risk population for T2DM. This affects the estimation of the effect size for the influence of mental disorders on the risk of developing the MetS. Further, we did not collect data on educational level and diet, and our physical activity assessment has not been validated. Similar instruments like the IPAQ-SF may overestimate the amount of physical activity (Lee et al. 2011). However, the study sample of 150 subjects is comprised of a consecutively assessed group of patients attending a diabetes prevention clinic at high risk for T2DM who were assessed with research diagnostic instruments and underwent oral glucose tolerance test. Moreover, since this was not a primary psychiatric sample, confounding through psychotropic medications was avoided.

## Conclusions

Our data in a high-risk group for T2DM support the relationship between depressive disorders and the MetS and point to a similar association with anxiety disorders. Future studies need to assess the bidirectional improvement in MetS and depression or anxiety disorders when one or the other or both aspects are treated. Furthermore, screening for depression and anxiety disorders is recommended for this population at risk for T2DM.
